# *Arabidopsis ANAC102*, Chloroplastic or Nucleocytosolic Localization?

**DOI:** 10.3390/genes14020438

**Published:** 2023-02-08

**Authors:** Alessandro Cresta, Stefano D’Alessandro

**Affiliations:** Dipartimento di Scienze della Vita e Biologia dei Sistemi, Università degli Studi di Torino, 10135 Turin, Italy

**Keywords:** chloroplast, retrograde signaling, signaling, *ANAC102*, NAC

## Abstract

*ANAC102* is a transcription factor involved in stress response and brassinosteroids signaling, with circadian regulation controlled by phytochromes. *ANAC102* has been proposed to have a role in downregulating chloroplast transcription, which may be very useful in reducing photosynthesis and chloroplast energy demand under stress conditions. However, its localization in the chloroplast has mainly been demonstrated by using constitutive promoters. In this work, we recapitulate the literature, clarify which are *ANAC102* isoforms in *Arabidopsis* and analyze their expressions under control conditions and in response to stress. Based on our results, the most highly expressed *ANAC102* isoform encodes for a nucleocytoplasmic protein and the N-terminal chloroplast-targeting peptide appears to be present only in Brassicaceae, and not involved in stress response.

## 1. Introduction

Photosynthetic organisms have been at the foundation of current life on Earth by providing oxygen and organic carbon. The photosynthesis reaction provides the chemical energy and reducing equivalents needed to fix atmospheric CO_2_. It is also highly regulated to be performed under fluctuating environmental conditions. Chloroplasts, the site of photosynthesis, play a major role in transmitting information about environmental conditions to the nucleus of the cell through a process known as retrograde signaling [1,2]. Several molecules, such as β-cyclocitral and 3′-phosphoadenosine 5′-phosphate (PAP), and proteins, such as Genome uncoupled 1 (GUN1) and EXECUTER1 (EX1), have been identified as key mediators of chloroplast-to-nucleus communication [3,4,5,6]. Retrograde signaling leads to a nuclear genetic response that is controlled by various transcription factors (TF), including the TGA, WRKY and NAC TF families [6,7,8].

The NAC domain protein family is one of the largest plant-specific transcription factor families, with 105 members in *Arabidopsis* thaliana [9]. The genes encoding these proteins, called *Arabidopsis* NAC DOMAIN-CONTAINING PROTEINS (ANACs), generate the NAM, ATAF and CUC (NAC) transcription factors that participate in a range of plant developmental processes. These proteins are characterized by the presence of an N-terminal NAC domain (NO APICAL MERISTEM (NAM), *Arabidopsis* ATAF1, ATAF2 and CUP-SHAPED COTYLEDON 2 (CUC2)) and a variable C-terminal transcriptional activation region [10,11]. In addition to their role in plant development, several ANACs are involved in response to stress conditions, such as ANAC019, ANAC055, ANAC072 (RESPONSIVE TO DESSICATION 26 (RD26)), ATAF1 and ATAF2 [12].

*ANAC102* has been observed to play a role in several pathways, and in this work we review its functions and present new data and a summary of the literature to shed light on its dual localization. 

## 2. Materials and Methods

### 2.1. Plant Growth Conditions and Treatments

*Arabidopsis* Columbia 0 (Col 0) seeds were used as wild type plants. For growth on plates, seeds were surface sterilized with 70% ethanol 0.01% Triton X-100, rinsed with 100% ethanol, dried and placed in culture plates containing 45 mL of 0.5× Murashige and Skoog salts [Caisson Labs], 0.5 g L^−1^ MES, and 0.8% Agar [Merck Sigma-Aldrich], adjusted to pH 5.7 with KOH. Plates were placed at 4°C for 2 days in the darkness, and then transferred to controlled growth conditions, with a 12 h/12 h photoperiod at 22 °C day/20 °C night. For non-sterile growth, *Arabidopsis thaliana* plants were grown in soil in a controlled environment at 150 µmol m^−2^ s^−1^ illumination, with a 12 h/12 h photoperiod at 22 °C day/20 °C night.

Three adult plants were placed in an air-tight glass dryer in presence of water or 100 µL βCC for 4 h at room temperature. Alternatively, seeds have been placed in an air-tight 12 × 12 cm squared culture plate sealed with Parafilm, placed at 4 °C for 2 days in darkness, and then transferred to controlled growth conditions.

### 2.2. RNA Preparation, cDNA Cloning and qRT-PCR Assays 

Total RNA was isolated and purified from the aerial part of three adult (5-week-old) *Arabidopsis* plants or from twenty 10-day-old seedlings by using TRIzol reagent (Thermo Fisher Scientific, Walthman, MA, USA). Sample quality and quantity were checked using the nanospectrophotometer BioSpec-nano (Shimadzu, Kyoto, Japan), according to manufacturer’s instructions.

Two micrograms of the obtained total RNA were retro-transcribed into cDNA with random primers and oligo(dT) using the High-Capacity cDNA Reverse Transcription Kit (Applied Biosystems, Foster City, CA, USA), according to the manufacturer’s recommendations.

The obtained cDNA was diluted 1:150 and used for qRT-PCR assays. All experiments were performed on a QuantStudio 3 Real-Time PCR System (Applied Biosystems, Foster City, CA, USA) using SYBR green I with ROX as an internal loading standard. The reaction was performed with a 15 μL mixture consisting of 7.5 μL 2X MaximaTM SYBR Green qPCR Master Mix (Thermo Fisher Scientific, Walthman, MA, USA), 0.5 μL cDNA and 250 nM primers. PCR conditions were the following for all primers: 10 min at 95 °C, 45 cycles of 30 sec at 95 °C, and 1 min at 60 °C. All runs were followed by a melting curve analysis from 55 to 95 °C. All amplification plots were analyzed with the MX3000PTM software to obtain Ct values. *PROFILIN1 (AT2G19760)* was used as a reference gene. 

Ct values were analyzed using the ΔCt method and statistical differences between conditions were evaluated. Primers used for real-time PCR were designed using the Primer-BLAST (http://blast.ncbi.nlm.nih.gov/Blast.cgi, accessed on the 15 December 2022) software and are shown in Supplementary Table S1.

### 2.3. Data Plotting and Statistical Analysis 

Graphs and statistical tests were generated in Python (Python Software Foundation, https://www.python.org/, accessed on the 15 December 2022) using the Panda [13], Matplotlib [14] and Seaborn [15] libraries. Statistical tests were performed using the Pingouin [16] library. A Games–Howell post hoc test was adopted for non-parametric data comparisons and a pairwise T-test using the Benjamini/Hochberg FDR correction for multiple comparisons of data with normal distributions. 

### 2.4. Protein Sequence Retrieval and Alignment

*ANAC102*.2 protein sequence was used as query in a blastp analysis (https://blast.ncbi.nlm.nih.gov/, accessed on the 15 December 2022) against the non-redundant protein sequences (nr) database using standard parameters and asking for a maximum of 5000 target sequences. Proteins showing a longer N-terminus have been selected and used for further analyses (KAF2580328.1, VDC93227.1, VDD27371.1, CAF1921732.1, CAH2043627.1).

Protein sequences have been aligned using Clustal Omega software (https://www.ebi.ac.uk/Tools/msa/clustalo/, accessed on the 15 December 2022) using standard parameters. 

## 3. Results

### 3.1. ANAC102, Chief or Teammate?

**Figure 1:**
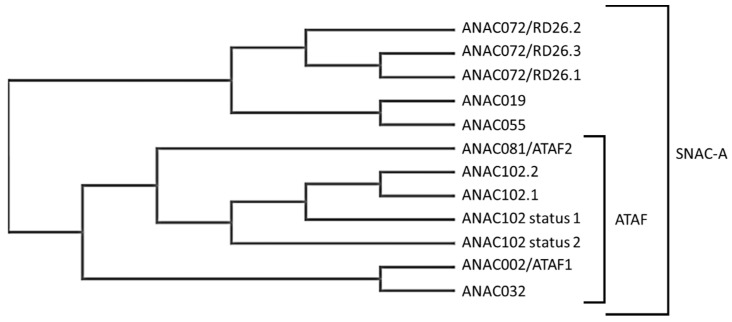
Guide tree of ANAC TFs of the SNAC-A subfamily [9,12].

*ANAC102* belongs to the ATAF subfamily of proteins that includes ANAC002/ATAF1, ANAC081/ATAF2 and ANAC032. The ATAF subfamily is a sister clade of ANAC072, ANAC019 and ANAC055, and the two clades constitute the stress-induced NAC-A (SNAC-A) family (Figure 1) [9,12].

Despite the high sequence conservation among *ANAC102*, ATAF1 and ATAF2 proteins, they likely have specific functions (Figure S1A) [9,17,18]. Indeed, we described a putative hierarchical regulation among these TFs in the regulation of the SCL14-dependent detoxification response under excessive light or in response to apocarotenoids [6,8]. However, the dominant behavior of *ANAC102* towards other members of the ATAF subfamily was not observed in response to the apocarotenoid phytohormone ABA [12]. Indeed, the response to ABA was still present in the single *ANAC102* knockout line, and only higher-order mutants were insensitive to ABA (the ABRE-binding proteins / ABRE-binding factors (AREB/ABF) quadruple mutant *anac002 x anac019 x anac055 x anac072* and the SNAC-A septuple mutant *anac002 x anac019 x anac032 x anac055 x anac072 x anac081 x ANAC102*) [12]. Recently, a direct interaction among *ANAC102*, ATAF1 and ATAF2 has been described and these results open a new possible interpretation in which the three ATAF-subfamily ANACs together might be necessary for downstream regulation rather than a hierarchical structure [19]. 

### 3.2. ANAC102 Is Involved in Several Pathways

*ANAC102* (*AT5G63790*) was first identified in 2005 as one of the most responsive genes to stress conditions, such as excessive light in the hydrogen peroxide over accumulating mutant line *cat2*, heat, drought and oxidative conditions [20].

Then *ANAC102* has been described in the response to low oxygen [21]. *ANAC102* was induced by waterlogging, both in roots and shoots of stressed *Arabidopsis*, and the mutant line was impaired in seed germination after flooding. 

Almost ten years later, we finally described one of the molecular mechanisms regulated by *ANAC102* in the response to excessive light [6]. Excessive light causes the accumulation of several toxic peroxides and metabolites, such as acrolein, and elicits a Scarecrow like 14 (SCL14)-dependent detoxification response, in which *ANAC102* regulates the induction of many detoxifying enzymes [8,22,23].

More recently, *ANAC102* has been found to play a role in brassinosteroids homeostasis by interacting with ANAC002/ATAF1, ANAC081/ATAF2 and Circadian Clock Associated 1 (CCA1) to regulate the expression of two cytochrome P450 enzymes, PHYB ACTIVATION TAGGED SUPPRESSOR 1 (BAS1) and SUPPRESSOR OF PHYB-4 7 (SOB7), which inactivate brassinosteroids (BRs) [19]. Brassinolide reduced *ANAC102* expression and the *ANAC102 Arabidopsis* mutant lines were partially insensitive to the phytohormone [19]. 

Finally, *ANAC102* has been shown to interact with both the Plastid Encoded Polymerase (PEP) and the Nuclear Encoded Polymerase (NEP) chloroplastic RNA polymerase, and its overaccumulation in the chloroplast led to the repression of chloroplastic genes [18].

### 3.3. ANAC102 Chloroplast or Nuclear?

**Figure 2:**
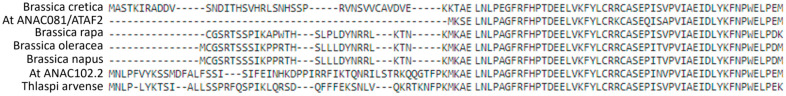
N’-terminus alignment of *ANAC102* and ATAF2 putative homologues from several representatives of the Brassicaceae family.

*ANAC102* has been described as a chloroplastic protein, a very intriguing feature for a TF which affects nuclear genes [18,24]. Its chloroplastic localization is driven by an 80 amino-acid N-terminal chloroplastic-targeting peptide (CTP), which has been extensively characterized [17,18]. The putative *ANAC102* protein possesses an N-terminal region, which is neither present in the very similar ATAF2 protein nor in other plants (Figure S1B) [18]. Repeating the analyses of the similarity of the *ANAC102*.2 protein by blastp highlighted the presence of a longer N-terminus in several plants of the Brassicaceae/Cruciferae family (Figure 2). This suggests that this domain may have appeared recently in *ANAC102*-like proteins, being present only in Brassicaceae, while ATAF2-like proteins are already present in Banana (*Musa paradisiaca*) [25] and can be found in Rosids and Monocots (blastp score > 290). 

*ANAC102* has been observed to accumulate extensively in the chloroplast or both in chloroplasts and nucleus in *Nicotiana benthamiana* and *Arabidopsis* when a constitutive promoter (35S CAMV) drives its expression [17,18,24]. Furthermore, preliminary data obtained through anti-GFP Western blotting of *ANAC102*-GFP overexpressing plants showed two bands that are likely due to the cleavage of the CTP of the chloroplast-addressed protein (63 kDa and around 58 kDa) [17].

At the same time, *ANAC102* regulates the expression of nuclear genes and it directly interacts with cytosolic proteins such as ANAC002/ATAF1, ANAC081/ATAF2 and CCA1 [6,8,19]. Indeed, it must colocalize with these proteins in the cytosol and in the nucleus. 

**Figure 3:**
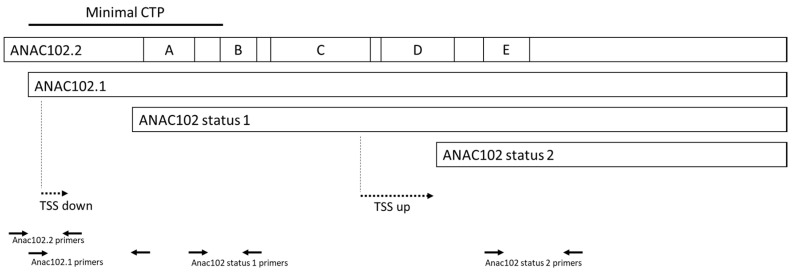
Schematic representation of *ANAC102* putative isoforms. NAC domains (A–E). Dashed arrows = Transcription starting site (TSS), black arrows = Primer location.

*ANAC102* has also been identified as a target of a phytochrome-dependent alternative promoter selection [26]. In this study, 2104 genes showed a pre-mRNA with different 5′ ends, indicating a different transcription starting site (TSS) in response to red light. Of these mRNAs, 1641 modifications led to a different N-terminus of the coded protein and 397 proteins showed a putative change in localization under red light [26]. *ANAC102* was not included among the proteins with a putative change in localization because all the TSSs identified in the study were downstream of the mRNA region coding for the CTP. The two proteins resulting from these TSSs, referred to as *ANAC102* status 1 (*ANAC102*s1) under normal light and *ANAC102* status 2 (*ANAC102*s2) under red light, cannot localize in the chloroplast. A schematic summary of the several putative isoforms of *ANAC102* is shown in Figure 3.

In agreement with these findings, the most N-terminal peptide of *ANAC102* identified in vivo, as listed on the *Arabidopsis* Information Resource (TAIR, www.arabidopsis.org accessed on the 15 December 2022) is at the beginning of *ANAC102* status 1 (Peptide PAp09544849). 

**Figure 4:**
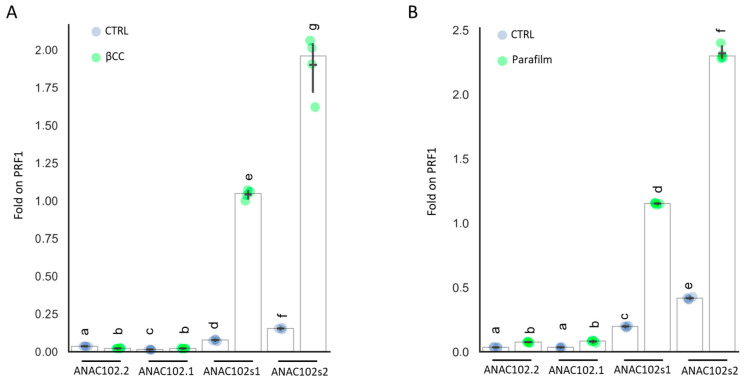
Expression of *ANAC102* isoforms under control conditions (CTRL), adult plants treated with β-cyclocitral (βCC) (**A**), or seedlings in Petri dishes sealed with parafilm (Parafilm). Results are presented as fold on PRF1 expression (**B**). White bars = median value, black dash = average. Error bar = confidence interval 95%. Data labeled with different letters are significantly different (*p* < 0.05).

To investigate the mRNA regulation of *ANAC102*, we prepared several primer pairs specific for the four possible isoforms, as shown in Figure 3, and assessed the expression of *ANAC102* isoforms by qRT-PCR. We tested two conditions known to induce *ANAC102* expression: a 4 h treatment with volatile β-cyclocitral (βCC) in adult (5 weeks) plants (Figure 4A) [27], and *Arabidopsis* seedlings grown in vitro in Petri dishes sealed with 3M tape as control or with Parafilm, to induce photorespiration (Figure 4B) [28,29]. 

We generated the template cDNA for the qRT-PCR reaction using both Oligo(dT) and Random Primers, to avoid 3′ enrichment bias. Indeed, the induction of *ANAC102* by βCC or by stress conditions had the same effect on the *ANAC102s1* and *ANAC102s2*, which were induced around 13 times by βCC and six times by the Parafilm treatment. This result shows that potential differences in retrotranscription efficiency between *ANAC102s1* and *ANAC102s2* do not affect the linearity of the response under our experimental conditions. Additionally, the distance between the *ANAC102.1* and *ANAC102s1* amplicons is small (50 bps) (Figure 3). 

Under control conditions, approximately 15% of total *ANAC102* transcripts encode for *ANAC102.2* and *ANAC102.1*, 30% for *ANAC102*s1 and 55% for *ANAC102*s2. This indicates that there may be a chloroplastic *ANAC102* protein, likely corresponding to *ANAC102.2*, in both young (10 days) and adult *Arabidopsis* plants. However, the majority (85%) of *ANAC102* RNA encodes for nucleocytoplasmic *ANAC102* isoforms. Both treatment with βCC and stress conditions consistently induced only the nucleocytoplasmic *ANAC102* isoforms, while *ANAC102.2* and *ANAC102.1* were repressed or only slightly induced. This response is likely phytochrome-independent, as it conserved the ratio between *ANAC102s1* and *ANAC102s2* transcripts.

It would be interesting to examine the expression of *ANAC102* isoforms in the *cat2* mutant line, which is the only condition in which the native *ANAC102* gene showed a chloroplastic localization [17].

Finally, previous studies have shown that the broad accumulation of *ANAC102* is dependent on new protein synthesis rather than the release from the chloroplast [6]. However, under certain conditions, such as a very strong oxidative stress induced by methyl viologen in the *cat2* mutant line, *ANAC102* may be released from the chloroplast [17]. It is worth noting that this release may occur only under extreme conditions that might compromise chloroplast integrity. 

## 4. Conclusions

*ANAC102* is a transcription factor that participates in stress response and brassinosteroids signaling, with a circadian regulation controlled by phytochromes. It has been shown to have a dual localization with the ability to be present in both the nucleus and the chloroplast, when expressed constitutively. In addition, *ANAC102* has been proposed to have a role in reducing chloroplastic transcription, which may be very useful for reducing photosynthesis and chloroplast energy demand under stress conditions. However, the *ANAC102* mutant line did not show any alteration in chloroplastic gene expression [18] and our results indicate that only the nucleocytoplasmic *ANAC102* isoforms are induced under stress conditions. 

Our results and the majority of literature support that the main functions of *ANAC102* are in cytosol and nucleus. This raises the question of whether the observed behavior of *ANAC102*, in repressing chloroplast gene expression when using a constitutive promoter, is a function present in nature. 

## Data Availability

The data presented in this study are available on request from the corresponding author.

## Supplementary Materials

The following supporting information can be downloaded at https://www.mdpi.com/article/10.3390/genes14020438/s1. Figure S1: Identity matrix and alignment of SNAC-A proteins; Table S1: Primer list.
