# Supplementary material for: Arabidopsis ANAC102, Chloroplastic or Nucleocytosolic Localization?
Source: Genes (Basel). 2023 Feb 8;14(2):438. doi: 10.3390/genes14020438 (PMC9956179; doi:10.3390/genes14020438)
Supplement: Supplementary file 1 [file genes-14-00438-s001.zip › Supplemental Table S1.pdf]

Supplemental table S1: Primer list

PRF1	AT2G19760	for	CCGGACTATTTCTCGGTGGC
PRF1	AT2G19760	rev	TGACACCTCCAGGTCCCTTC
ANAC102.2	AT5G63790	for	tccccttcgtttataaaagctccA
ANAC102.2	AT5G63790	rev	TCGAATCGGAGGATCTTTGTGG
ANAC102.1	AT5G63790	for	GGACTTTGCTCTCTTCTCCTCG
ANAC102.1	AT5G63790	rev	GGCAAATTCAACTCCGCCTT
ANAC102 status 1	AT5G63790	for	CGGAGATGTGCGTCAGAACC
ANAC102 status 1	AT5G63790	rev	ACAACGCCATTTCTGGAAGCT
ANAC102 status 2	AT5G63790	for	GAACAACTTAAGACTTGATGATTGGGTTTTGT
ANAC102 status 2	AT5G63790	rev	TTGAGCATCTTGAGTCCGACG
