# Supplementary figures and images for: Arabidopsis ANAC102, Chloroplastic or Nucleocytosolic Localization?
Source: Genes (Basel). 2023 Feb 8;14(2):438. doi: 10.3390/genes14020438 (PMC9956179; doi:10.3390/genes14020438)

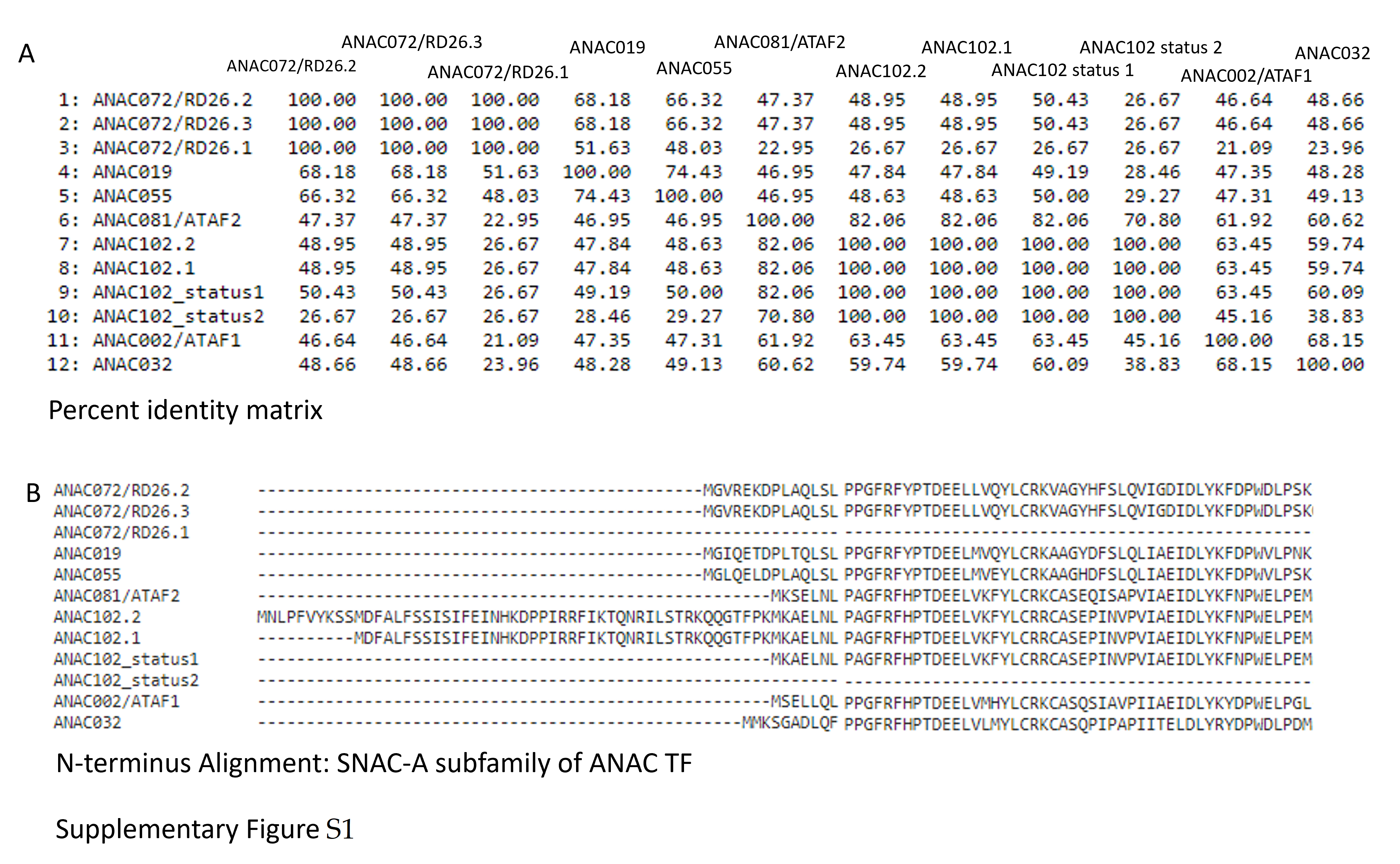

Supplement: Supplementary file 1 [file genes-14-00438-s001.zip › Supplemental Figure S1.tif]
